# Complete Genome Sequence of Iris Severe Mosaic Virus Isolated in Japan

**DOI:** 10.1128/MRA.00093-19

**Published:** 2019-04-11

**Authors:** Masanobu Nishikawa, Ryosuke Tokuda, Tetsuya Yoshida, Takamichi Nijo, Noriko Maruyama, Kosuke Katsu, Kensaku Maejima, Yasuyuki Yamaji, Shigetou Namba

**Affiliations:** a Department of Agricultural and Environmental Biology, Graduate School of Agricultural and Life Sciences, The University of Tokyo, Tokyo, Japan; KU Leuven

## Abstract

The complete genome sequence of an iris severe mosaic virus isolate (ISMV) from Iris tectorum in Japan was determined for the first time. According to sequence identity analyses, our specimen is closely related to isolates reported from China.

## ANNOUNCEMENT

Iris severe mosaic virus (ISMV) is a member of the genus Potyvirus in the family Potyviridae and possesses flexuous filamentous particles and a single-stranded positive-sense RNA genome (1). ISMV has been detected in plants in the family Iridaceae (2). ISMV causes chlorotic stripes and mosaic symptoms on the leaves and color breaking on the petals. Heavily infected plants exhibit stunting and distortion of leaves, resulting in the death of plants and reduction of commercial value (3). To date, the complete genomes of two ISMV isolates from China and the partial sequences of several ISMV isolates from China, South Korea, India, Iran, Mexico, and the Netherlands have been reported (4). Although ISMV has been detected in Japan (5), no sequence information for Japanese ISMV isolates has been reported. Here, we report the first complete genome sequence of ISMV isolated in Japan.

In 2018, *Iris tectorum* leaves showing mosaic symptoms were collected in Tokyo, Japan. Total RNA was extracted from leaves using the cetyltrimethylammonium bromide method (6) and treated with DNase I (Nippon Gene, Japan). A paired-end sequencing cDNA library was constructed from the extracted RNA using the NEBNext Ultra II RNA library prep kit for Illumina (New England Biolabs, USA), according to the manufacturer’s instructions except for poly(A)-tailed mRNA purification, and sequenced using a MiSeq instrument (Illumina, USA) and the MiSeq reagent kit v.2 (500 cycles). A total of 1,459,698 paired-end reads were obtained. After quality control and adapter trimming using Trimmomatic v.0.36 software (7) (ILLUMINACLIP:adapters.fa:2:30:10, LEADING:20, TRAILING:20 SLIDINGWINDOW:4:15, MINLEN:36, and other parameters with default settings), the reads were *de novo* assembled using Trinity v.2.6.6 software (8) with default settings. A BLASTn search (9) of the assembled contigs against the GenBank database revealed that 16 contigs with a total length of 7,370 bp showed sequence identity with ISMV isolates (GenBank accession numbers KT692938 and MF385582). The obtained contigs covered 70% of the ISMV isolate genome represented by GenBank accession number MF385582, and 10 regions between contigs and the 5′ and 3′ terminal regions were undetermined. To determine the complete genome sequence of the Japanese isolate of ISMV (ISMV-J), undetermined regions between contigs were amplified by reverse transcription-PCR (RT-PCR), using pairs of ISMV-specific primers, and sequenced. The undetermined 5′ and 3′ terminal fragments were amplified using the 5′ rapid amplification of cDNA ends (RACE) system kit v.2 (Invitrogen, USA) and RT-PCR with ISMV-specific and oligo(dT) primers, respectively. The amplified fragments were cloned into pCR-Blunt II-TOPO vector (Invitrogen), and 4 and 8 clones of the 5′ and 3′ terminal regions, respectively, were sequenced.

The complete genome sequence of ISMV-J was 10,403 nucleotides (nt) long, excluding the poly(A) tail at its 3′ end, with a GC content of 39.2%. Like other potyviruses, a large open reading frame (ORF) (nt 124 to 10074) was predicted in the ISMV-J genome using the NCBI ORFfinder. The large ORF was predicted to encode a 377-kDa polyprotein with nine potential potyvirus cleavage sites, as in the case of a previously reported ISMV isolate (GenBank accession number KT692938). P3N-PIPO, an ORF conserved throughout the family Potyviridae (10) and translated by a transcriptional slippage at GA_6_ (nucleotides 3640 to 3646), was also found at the same region, as in the case of a previously reported ISMV isolate (GenBank accession number KT692938). The sequence alignments with reported ISMV isolates were performed using the MUSCLE algorithm (11) in the program Sequence Demarcation Tool (SDT) v.1.2 (12) with default settings. The percent identities of the large ORF with those of previously reported ISMV isolates (GenBank accession numbers KT692938 and MF385582) were 92.1% and 92.7% at the nucleotide level and 96.0% and 96.4% at the amino acid level, respectively. Based on the current classification criteria of the family *Potyviridae* (1), ISMV-J was proven to be with that of an isolate of ISMV. The coat protein shared higher amino acid sequence identity (98% to 100%) with isolates from China than with isolates from other countries (85% to 87%). These results suggest that ISMV-J is closely related to ISMV isolates reported from China.

### Data availability.

The genome sequence of ISMV-J was deposited in the DNA Data Bank of Japan under accession number LC433737. The raw sequence data were deposited in the National Center for Biotechnology Information Sequence Read Archive (SRA) under BioSample number SAMN11053879 and SRA run number SRR8668529, which are part of the SRA study number PRJNA525575.
